# Congenital Insensitivity to Pain: A Case Report and Review of the Literature

**DOI:** 10.1155/2014/141953

**Published:** 2014-09-18

**Authors:** Leema Reddy Peddareddygari, Kinsi Oberoi, Raji P. Grewal

**Affiliations:** ^1^The Neuro-Genetics Institute, 501 Elmwood Avenue, Sharon Hill, PA 19079, USA; ^2^Neuroscience Institute, Saint Francis Medical Center, School of Health and Medical Sciences, Seton Hall University, Saint Francis Medical Center, 601 Hamilton Avenue, Trenton, NJ 08629, USA

## Abstract

Congenital insensitivity to pain (CIP) is a rare autosomal recessive genetic disease caused by mutations in the *SCN9A* gene. We report a patient with the clinical features consistent with CIP in whom we detected a novel homozygous G2755T mutation in exon 15 of this gene. Routine electrophysiological studies are typically normal in patients with CIP. In our patient, these studies were abnormal and could represent the consequences of secondary complications of cervical and lumbosacral spine disease and associated severe Charcot's joints.

## 1. Introduction

Autosomal recessive congenital insensitivity to pain (CIP) is a rare condition, affecting very few individuals, but with a worldwide distribution. CIP is clinically characterized by the ability to feel a given stimulus but also the inability to perceive pain. This is in contrast to congenital “indifference” to pain which implies a lack of concern to a painful stimulus that is received through normal sensory pathways and may be associated with central nervous system disorders such as schizophrenia or pervasive development disorder [1].

CIP is genetically and clinically heterogeneous caused by mutations in several different genes. For example, mutations in the* neurotrophic tyrosine kinase receptor type 1 gene (NTRK1)* and* nerve growth factor-*β* (NGFB)* result in CIP with an anhidrosis phenotype [2, 3]. In contrast, homozygous loss of function mutations in* sodium channel voltage-gated type IX, alpha subunit (SCN9A)* gene has been reported to result in the CIP with an anosmia phenotype [4]. Although this condition is rare, genotype phenotype studies of such patients are important.

We report the results of our analysis of a patient who we encountered in our neurology clinic with a history of insensitivity to pain.

## 2. Case Report

This 58-year-old woman presented with a long history of insensitivity to pain since childhood and increased numbness in her legs for several years. As a child, she recalled developing cuts on her feet that she could not feel. She could distinguish between hot and cold temperature although there was no uncomfortable sensation associated with extremes of either one. Since the age of 15 years she started to develop frequent fractures involving multiple bones which were also painless. In addition, she has two children and suffered no pain during childbirth. She also had anosmia. Over the ten years prior to evaluation, she had started to develop sensory loss in her legs. She had previously been diagnosed with cervical and lumbar spine disease and had undergone surgical treatment of both of these regions of her spine. She is of Caucasian English descent and the product of a nonconsanguineous marriage. She has a healthy brother and two healthy children. There is no indication that either her parents or any other relative was affected by symptoms suggestive of CIP suggesting an autosomal recessive form of inheritance. The remainder of the general medical history was significant for absence of diabetes, cancer, or rheumatologic disease. Neurological examination revealed normal mental status and cranial nerve examination except for anosmia. She was diffusely areflexic with flexor plantar responses. She had multiple joint deformities involving both ankles, elbows, and knees (Charcot's joints) which limited the testing of power. When she could provide a good effort, she had good strength. She had decreased sensation to pin prick, proprioception, and vibration distally in her feet. She could not perform a tandem walk and had a positive Romberg's test.

An electromyogram (EMG) was performed; the motor nerve conduction parameters were normal in the right tibial nerve but showed a markedly reduced response amplitude in the right peroneal nerve recording the extensor digitorum brevis muscle (this was severely atrophied). No evoked response could be elicited with stimulation of the peroneal nerve at the fibular head. No evoked sensory nerve action potentials were obtained in the right ulnar, sural, and superficial peroneal nerves. Needle electromyogram showed no abnormal spontaneous activity in any muscle sampled and the presence of high amplitude polyphasic units in the distal muscles of the right arm and legs associated with a mildly reduced interference pattern with maximal effort. Overall the study was interpreted as showing chronic neurogenic changes with a superimposed entrapment neuropathy of the right ulnar nerve. The other abnormalities noted in the nerve conductions were interpreted as partly secondary to Charcot's joints and technical factors such as increased subcutaneous tissues.

## 3. Genetic Analysis

Following IRB approved policies and procedures, a blood sample was obtained and DNA was extracted. Whole exome sequencing was performed by commercial sequencing company. Exome capture was performed by HiSeq2000 using a paired-end (2 × 100) protocol, Illumina raw data processing, and Agilent SureSelect exome kit for exome enrichment. The sequences were aligned to human genome reference (UCSC version hg 19). Nucleotide-level variation analysis of the exome sequence data was performed using the DNA nexus platform (https://dnanexus.com/). The variants obtained with this platform were further annotated using Ensembl variant effect predictor tool (Ensembl release 75, February 2014) (http://useast.ensembl.org/info/docs/tools/vep/index.html) [5]. Since CIP is a rare disorder, the minor allele frequency was assigned at less than 1%. These results were further filtered for homozygous, nonsynonymous variants with deleterious, possible damaging and unknown effect using SIFT and Polyphen analysis. This narrowed down the list of variants to 584.

**Figure 1:**
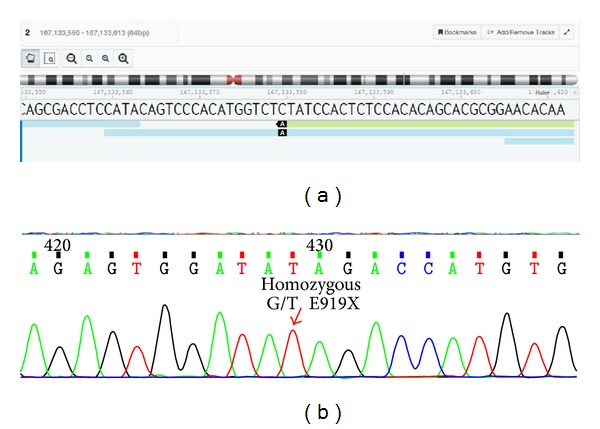
Image showing the homozygous variant on chromosome 2. (a) Image identifying the homozygous A/A mutation on chromosome 2 at position 167133579 using the DNA nexus platform. (b) Image showing the homozygous c. G2755T mutation in exon 15 of* SCN9A* gene following amplification and Sanger's sequencing.

Those single nucleotide polymorphisms (SNPs) involving genes known to cause insensitivity to pain were then analyzed. A potentially significant variant was identified on chromosome 2 at position 167133579, a homozygous A/A variant (Figure 1(a)). This homozygous c. G2755T mutation in exon 15 of* SCN9A* gene results in a stop mutation, causing premature truncation of the protein p. E919X. This SNP was reconfirmed by amplification and Sanger's sequencing (Figure 1(b)).

## 4. Discussion

The* SCN9A* gene is expressed in all sensory neurons and is a key molecule in the processing of peripheral pain. This gene encodes a voltage-gated sodium channel (Nav 1.7) which plays a significant role in nociceptive signaling and both gain and loss of function mutations have been reported. Interestingly, depending upon the specific mutation, there is a marked diversity of resulting phenotype. For example, gain of function mutations causes inherited erythromelalgia and paroxysmal extreme pain disorder which follow an autosomal dominant pattern of inheritance [6, 7]. More recently there have been reports of mutations causing seizures or a small fiber neuropathy [8, 9].

Studies in individuals with CIP from seven different populations identified homozygous mutations in* SCN9A* gene [10]. Loss of function mutations in* SCN9A *gene causes truncation of the encoded sodium channel Nav 1.7 protein, resulting in channelopathy-associated autosomal recessive congenital insensitivity to pain. Twenty-seven different* SCN9A* gene mutations have been reported in CIP patients so far (Table 1). Given the predicted consequences of the novel change in the* SCN9A* gene in our patient, it is likely to be a disease producing mutation and brings the total number of mutations to twenty-eight.

Although the primary consequence of the homozygous* SCN9A *mutation is the absence of pain sensation, there are associated conditions including anosmia, self-mutilation resulting in oral and digit lesions, multiple injuries due to repeated trauma, burn-related injuries, orthopedic complications that include bone deformities from untreated fractures, osteomyelitis, and neuropathic joints later in life [4, 8, 9, 11–14]. Although Charcot's joints are commonly reported in patients with CIP, bony involvement of the spine as seen in our patient is rare; however, anosmia and Charcot's joints noted in our patient are comorbidities that were previously reported associated with CIP [4, 8, 9, 14].

Routine EMG studies of patients with CIP are typically normal. In our patient, it is likely that the abnormalities detected on both the nerve conduction studies and needle examination are secondary to cervical and lumbosacral spine disease, joint deformities, and muscle wasting associated with Charcot's joints. However, a sensory motor peripheral neuropathy is not excluded by this examination. It is possible that the patient has an associated large fiber neuropathy which may be related to the G2755T mutation or alternatively to another unrelated etiology. A possible relationship between mutations in the SCN9A and a large fiber neuropathy could be supported by genotype/phenotype analysis in further patients with CIP.

The study of our patient expands the spectrum of mutations that have been reported to cause this disorder. In addition, our analysis demonstrates the power of next generation sequencing that can enable genetic confirmation of a suspected diagnosis of a rare disorder.

## Figures and Tables

**Table 1:** *SCN9A* mutations causing congenital indifference to pain.

*SCN9A* mutations	Reference
c.1376C>G, p.Ser459Ter	Cox et al., 2006 [4]
c.2298delT, pIle767Ter	
c.2691G>A, p.Trp897Ter	

c.828delGT	Nilsen et al., 2009 [9]
c.2575C>T	

c.829C>T, p.Arg277Ter	Goldberg et al., 2007 [10]
c.984C>A, p.Tyr328Ter	
c.2455C>T, p.Arg630X	
c.3600delT, p.Phe1200LeufsX33	
c.4462C>T, p.Arg1488X	
c5067G>A, p.Trp1689X	
c.2076_2077InsT, p.Glu693X	
c.4366-7_10delGTTT, del 4 bp, splice junction mutation	
c.3703_3713del, del 11 bp, pIle1235LeufsX2	
c.4975A>T, p.Lys1450X	

c.1126A>C, p.K376Q	Shorer et al., 2014 [14]
c.1124delG, p.G375AfsX5	

c.984C>A, p.Y328X	Ahmad et al., 2007 [15]

c.2687G>A, p.R896Q	Cox et al., 2010 [16]
c.4108_4122delCGATGGAAAAACCTG, p.R1370-L1374 del	
c.4474delA, p.I1493SfsX8	

c.1567C>T, p.Arg523Ter	Kurban et al., 2010 [17]

c.2697G>A, p.Met899Ile	Yuan et al., 2011 [18]
c.2796A→C, p.Met932Leu	

c.5155T>C; C1719R	Staud et al., 2011 [19]
c.3467+3delA, or IVS17+3 delA	

c.1567C>T, p.Arg523Ter	Klein et al., 2013 [20]
IVS8-2A>G	
